# Application of imputation methods to genomic selection in Chinese Holstein cattle

**DOI:** 10.1186/2049-1891-3-6

**Published:** 2012-02-29

**Authors:** Ziqing Weng, Zhe Zhang, Xiangdong Ding, Weixuan Fu, Peipei Ma, Chonglong Wang, Qin Zhang

**Affiliations:** 1 Key Laboratory of Animal Genetics Breeding and Reproduction, Ministry of Agriculture, College of Animal Science and Technology, China Agricultural University, Beijing, China

**Keywords:** Chinese Holstein Cows, dairy cattle, genomic selection, imputation methods, quality control, SNP

## Abstract

Missing genotypes are a common feature of high density SNP datasets obtained using SNP chip technology and this is likely to decrease the accuracy of genomic selection. This problem can be circumvented by imputing the missing genotypes with estimated genotypes. When implementing imputation, the criteria used for SNP data quality control and whether to perform imputation before or after data quality control need to consider. In this paper, we compared six strategies of imputation and quality control using different imputation methods, different quality control criteria and by changing the order of imputation and quality control, against a real dataset of milk production traits in Chinese Holstein cattle. The results demonstrated that, no matter what imputation method and quality control criteria were used, strategies with imputation before quality control performed better than strategies with imputation after quality control in terms of accuracy of genomic selection. The different imputation methods and quality control criteria did not significantly influence the accuracy of genomic selection. We concluded that performing imputation before quality control could increase the accuracy of genomic selection, especially when the rate of missing genotypes is high and the reference population is small.

## Background

Genomic selection is becoming prevalent and practicable in dairy cattle breeding, where genomic breeding values of animals are estimated using high density single nucleotide polymorphisms (SNPs) and are the basis for the selection of elite animals [1]. Genomic selection combines information on genotypes, phenotypes and pedigree to increase the accuracy of the estimated breeding values (EVBs). Low-, medium- and high-density platforms have become available and this new technology has revolutionized dairy cattle breeding and has led to an extraordinary amount of research activity [2-4]. Tens of thousands of dairy cattle have been genotyped using the BovineSNP50 BeadChip (Illumina Inc. San Diego, CA) or related platforms, and the resulting genomic data have been evaluated [5]http://www.interbull.org/. Genomic estimated breeding values (GEBVs) are at the core of genomic selection. The GEBV is calculated as the sum of all SNP effects; the estimation of SNP effects therefore plays an important role in genomic selection. In the SNP genotype data obtained from the SNP chip technique, missing genotype information is a common phenomenon that leads to a low call rate for some SNPs and for some animals. The routine data quality control procedure in genomic selection eliminates SNPs and animals with low call rates from the data sets, resulting in the loss of information and a decrease in the accuracy of the GEBV. Imputation can be used to deduce the missing genotypes and could be helpful in increasing the accuracy of genomic selection. Imputation also allows for the use of low-density chips that may be more cost-effective, facilitating the widespread implementation of whole-genome selection [5,6].

Several imputation methods have been proposed and are implemented in programs like fastPHASE [7], Beagle [8], and findhap [9]. These methods impute the missing genotypes based on reconstructed haplotypes informed by linkage disequilibrium between SNPs. They all use different methods of haplotype reconstruction which leads to differences in the accuracy of estimated genotypes and different computing time. FastPHASE and Beagle run slowly as Bayesian method are applied for haplotype reconstruction, which may limit their practical use in large data sets. findhap runs very fast and is comparable with fastPHASE and Beagle in accuracy [5].

In genomic selection, quality control of the SNP data is a necessary step before the SNP effects are estimated [10,11]. Two alternative strategies can be considered; imputation before or imputation after quality control. The optimal quality control criteria might be different with and without imputation. The objective of the present study is to evaluate the efficiency of different imputation strategies in Chinese Holstein Cattle based on the accuracy of genomic selection. We varied the imputation strategies, the criterion of quality control, and the order of imputation with quality control, to find out whether these elements affect the accuracy of the genomic predictions.

## Materials and methods

### Data

A total of 2, 180 Chinese Holstein Cattle (87 bulls and 2, 093 cows) born between 2001 and 2006 were genotyped for 54, 001 SNP markers distributed across the 29 *Bos taurus *autosomes and the × chromosome using the Illumina Bovine SNP50TM chip (Illumina Inc., San Diego, CA, USA). The 2, 093 cows formed the reference population. Their 13 sires were used for imputation of the missing SNP genotypes. The average number of daughters per sire was 161; the range was from 83 to 358. The cows came from 14 Holstein cattle farms in Beijing, China, where regular and standard performance testing (Dairy Herd Improvement, DHI) has been conducted since 1999 [12]. The remaining 74 bulls were used as the validation population. All cows and bulls had official EBVs for milk production traits that were provided by the Dairy Association of China. The EBVs were obtained using the DHI data for the whole Chinese Holstein population and the conventional BLUP method based on a multiple trait random regression test-day model [13]. The bulls in the validation population were all proven bulls with the average reliability of EBVs greater than 0.90. In this study, the EBVs of three milk production traits of the cows, milk yield (MY), fat percentage (FP) and protein percentage (PP), were used to estimate the SNP effects that were then used to calculate the GEBVs of the bulls in the validation population. The heritabilities of the three traits were estimated based on the whole Chinese Holstein population using the DMU software [14]. Detailed descriptions of the traits and their EBVs are given in Table 1.

**Table 1:** Descriptive statistics and accuracies of the EBVs of three milk production traits

Traits	Mean (range)	Standard deviation	Mean reliability (range)
Milk yield	379.36 (-1, 667.00 to 2, 552.00)	608.65	0.63 (0.50 to 0.71)

Fat percentage	-0.07 (-0.90 to 0.91)	0.27	0.52 (0.41 to 0.70)

Protein percentage	-0.01 (-0.42 to 0.32)	0.10	0.52 (0.41 to 0.70)

### Imputation and quality control strategies

#### Imputation method

Two imputation methods were implemented: 1) the findhap program v1 [9] was used to impute missing SNP genotypes using estimated genotypes (Impute A); 2) missing genotypes were directly replaced with the heterozygote (Impute B).

#### Quality control

Three criteria of minor allele frequency (MAF) of SNPs were considered, > 3% (QC3%), > 5% (QC5%) and > 0% (QC0), for marker filtration. For all of the three criteria, the SNP call rate and individual call rates were required to be more than 90%.

To study the order of imputation and quality control, six strategies were designed (see Table 2) and their accuracy was compared with the GEBVs in the validation population. For all six strategies, the SNP data were first checked for Mendelian errors and genotypes with Mendelian errors were deleted and treated as missing.

**Table 2:** Datasets in the reference population generated using five different imputation and quality control strategies

Strategy^1^	No. of animals	No. of SNPs
Imputation before quality control		

S1: Impute A - QC3%	2, 092	45, 072

S2: Impute B - QC3%	2, 092	45, 614

S3: Impute A - QC5%	2, 092	43, 710

S4: Impute A - QC0	2, 092	53, 973

Imputation after quality control		

S5: Impute A - QC3%	2, 021	43, 481

S6: Impute A - QC5%	2, 021	41, 866

^1^Impute A, imputation with findhap v1 [9]; Impute B, missing genotypes were directly replaced with heterozygote.

Abbreviations: QC3%, SNP MAF > 3%; QC5%, SNP MAF > 5%; QC0, no requirement for MAF.

### Estimation of SNP effects

The BayesB method [1] was adapted to estimate the SNP effects using the SNP data resulting from each of the five imputation and quality control strategies and the EBVs of cows in the reference population. The statistical model of BayesB is as follows:

(1) $$Y=\mathrm{Xb}+\overset{N}{\underset{i=1}{\sum }}Z_{i}g_{i}+e$$

where **Y **is the vector of the EBVs for MY, FP or PP, **b **is a vector of fixed effects, **g_i _**is the i^th ^marker effect, ***N ***is the total number of markers, **X **and **Z **are design matrices corresponding to **b **and **g**, and **e **is a vector of residual errors. The design matrix **Z **contains indicator variables 0, 1 and -1 corresponding to the SNP genotypes 12, 22 and 11, respectively. It was assumed that residuals **e **were independent and follow a normal distribution, **e **~ *N *(0, σ_e_^2^). All marker effects **g_i _**were also assumed to be normally distributed, **g_i _~ *N *(0, σ_gi_^2^)**. The marker effects were assumed to be zero with a probability of π or non zero with a variance that followed a scaled inverse chi-square distribution with a probability of (1 - π) [1].

In this study, the prior probability π was assumed to be 0.95 based on the results of Zhang et al. [15], BayesB was implemented with the Monte Carlo Markov Chain (MCMC) algorithm, which is a mixture of Gibbs sampling and Metropolis-Hastings sampling as described by Meuwissen et al. [1]. The MCMC chain was run for 10, 000 cycles with 100 cycles of Metropolis-Hastings sampling within each Gibbs sample. The first 2, 000 cycles were discarded as burn-in. All the samples of SNP effects from later cycles were averaged to obtain the estimate of marker effects.

### GEBV calculation and accuracy of GEBVs

The GEBVs for the bulls in the validation population were calculated as the sum of all estimated SNP effects according to their marker genotypes [1]. The accuracies of the GEBVs were measured as correlations between the GEBVs and the known conventional EBVs.

## Results

The original SNP dataset for the reference population consisted of the genotypes of 54, 001 SNPs for the 2, 093 cows. The total proportion of missing genotypes in this population was 1.92%. The call rates for the SNPs ranged from 0% to 100% with an average of 97.98%. The call rates for the animals ranged from 70.27% to 99.43% with an average of 98.05%; one cow for which the call rate was extremely low (43.52%) was discarded from the dataset. The proportions of animals and SNPs with call rate less than 90% were 3.01% and 2.18%, respectively. The proportions of SNPs with MAFs less than 0.03 and less than 0.05 were 17.23% and 20.53%, respectively. Of the SNPs with call rates less than 90% (average 67.83%), the average MAF was 0.165 and the proportions with MAFs of less than 0.03 and less than 0.05 were 26.06% and 35.89%, respectively. For the SNPs with call rates greater than 90% (average 98.77%), the average MAF was 0.228 and the proportions with MAFs less than 0.03 and less than 0.05 were 17.03% and 20.18%, respectively. This result indicates that SNPs with low call rates tend to have low MAFs. After data editing using the six imputation and quality control strategies, six different SNP datasets were generated (Table 2). Strategies using imputation before quality control (S1, S2, S3 and S4) produced larger data sets with slightly more animals and many more SNPs than strategies using imputation after quality control (S5 and S6). After imputation, the call rates or all SNPs and all animals was as high 100%. Using Impute A (Impute B), the proportions of SNPs with MAF < 0.03 and ≤ 0.05 deceased to 16.53% (15.53%) and 19.06% (19.06%), respectively. The data sets generated using strategies S1, S2, S3 and S4 contained the same number of animals, but different numbers of SNPs (the data set from S2 contained 542 more SNPs than the data set from S1 and 1, 904 more than the data set from S3). The strategy without the requirement for MAF (S4) retained almost all SNPs after imputation (only 28 SNPs were excluded because they are heterozygous in all individuals and thus non-informative). Similarly, the data sets generated from S5 and S6 contained the same number of animals, but S5 retained 1, 615 more SNPs than S6. Overall, S4 yielded the largest data set and S6 the smallest one.

The estimated heritabilities and accuracies of the GEBVs obtained using the different datasets arising from the different imputation and quality control strategies are shown in Table 3. For all the strategies, the accuracy increased along with heritability. The accuracies from the four strategies with imputation before quality control (S1, S2 S3 and S4) were around 1% to 2% higher than those from the two strategies with imputation after quality control (S5 and S6). The performances of S1, S2 and S3 were similar and slightly better than S4. S5 performed slightly better than S6.

**Table 3:** Estimated heritabilities (h^2^) and accuracies of the GEBVs measured as correlations between the GEBVs and the conventional EBVs in the validation population using different imputation and quality control strategies ^1^

Trait	h^2^	Accuracy					
		
		S1	S2	S3	S4	S5	S6
Milk yield	0.36	0.65	0.65	0.65	0.64	0.64	0.63

Fat percentage	0.41	0.74	0.74	0.74	0.74	0.73	0.72

Protein percentage	0.23	0.58	0.58	0.58	0.56	0.57	0.56

^1^S1-S6 represent the different imputation and quality control strategies as described in the footnotes to Table 2.

## Discussion

The number of individuals in the reference population and the number of SNPs in the genome with known genotypes are the two major factors affecting the accuracy of the predicted GEBVs [16]. Missing genotype information is a common feature of high density SNP datasets which, after data quality control, reduces the number of available SNPs as well as the number of individuals available for estimating SNP effects. For example, a reference population of 798 Australian Holstein-Friesian bulls with known genotypes of 56, 947 SNPs was reduced to 730 bulls with 38, 259 SNPs after quality control [16]. The same phenomenon was observed in the present study. Several methods exist to deal with missing genotypes. The simplest way is to impute the missing genotypes with heterozygotes. A more realistic method is to impute the missing genotypes with estimated genotypes based on reconstructed haplotypes informed by linkage disequilibrium between SNPs. In addition to selecting the imputation method, the criteria used for quality control needs to be considered and a decision about whether to perform imputation before or after data quality control needs to be made. In this study, we compared six imputation and quality control strategies using a real dataset. Our results showed that the strategies with imputation before quality control could bring the call rates of all SNPs and animals to 100%, and increase the MAF of the SNPs; so, fewer animals and SNPs were excluded from the original dataset as a result of quality control. Because of the larger dataset that can be generated, the accuracies of the GEBVs from the strategies with imputation before quality control were higher than those from the strategies with imputation after quality control. However, MAF was still a factor that affected the accuracy of the GEBVs. Our results showed that the strategy without a requirement for MAFs underperformed compared with those with a requirement for MAF > 0.03 or > 0.05, although the former strategy produced the largest dataset.

It should be noted that the two imputation methods, Impute A and Impute B, resulted in almost the same size of dataset (Impute B gave slightly more SNPs) and the same accuracies of the GEBVs. The likely reason for the high similarity of the two datasets after imputation is that the missing SNP rate in the original dataset was relatively small (1.92%) and the majority of the missing genotypes were probably real heterozygotes. Indeed, the two datasets shared 99.13% identical SNP genotypes in each individual. When the missing SNP rate is high, based on our result, it is reasonable to suppose that Impute A would result in a higher accuracy for the GEBVs because this method imputes the missing genotypes more accurately.

In this study, two criteria for MAF, > 3% and > 5% were applied for quality control either after or before imputation. Obviously, with the stricter criterion for MAF ( > 5%) more SNPs would be excluded from the dataset. Our results showed that, when imputing before quality control, the dataset with MAF > 3% contained 1, 362 more SNPs than the dataset with MAF > 5%; when imputing after quality control, the dataset with MAF > 3% contained 1, 615 more SNPs than the dataset with MAF > 5%. This difference is because when imputing before quality control, the MAF of the SNPs would increase along with the increased call rate as a result of imputation, so that some SNPs with MAF < 5% in the original dataset become > 5% after imputation. Our results show that, when imputing before quality control, there is no difference in the accuracies of the predicted GEBVs from datasets with MAF > 3% and MAF > 5% for all traits; when imputing after quality control, the accuracies from the dataset with MAF > 3% are slightly higher than those from the dataset with MAF > 5%. This observation suggests that using MAF > 3% is more appropriate than using MAF > 5%.

In conclusion, we found that conducting imputation before quality control could be a useful strategy to increase the accuracy of genomic selection, especially when the rate of missing genotypes is high and the reference population is small.

## Competing interests

The authors declare that they have no competing interests.

## Authors' contributions

ZW carried out the design of the study, participated in the statistical analysis and drafted the manuscript. ZZ carried out the statistical analysis. WF participated in the design of the study and statistical analysis. PM and CW participated in the statistical analysis. QZ and XD conceived of the study, and participated in its design and coordination. All authors read and approved the final manuscript.

## References

[B1] Meuwissen THE Hayes BJ Goddard ME Prediction of total genetic value using genome-wide dense marker maps Genetics 2001 157 1819 1829 11290733 10.1093/genetics/157.4.1819 PMC1461589

[B2] de Roos APW Schrooten C Mullaart E Calus MPL Veerkamp RF Breeding value estimation for fat percentage using dense marker on *Bos taurus *autosome 14. J Dairy Sci 2007 90 4821 4829 10.3168/jds.2007-0158 17881705

[B3] VanRaden PM Efficient methods to compute genomic predictions J Dairy Sci 2008 91 4414 4423 10.3168/jds.2007-0980 18946147

[B4] VanRaden PM Van Tassell CP Wiggans GR Sonstegard TS Schnabel RD Taylor JF Schenkel FS Invited review: Reliability of genomic predictions for North American Holstein bulls J Dairy Sci 2009 92 16 24 10.3168/jds.2008-1514 19109259

[B5] Weigel KA Van Tassell CP O'Connell JR VanRaden PM Wiggans GR Prediction of unobserved single nucleotide polymorphism genotypes of Jersey cattle using reference panels and population based imputation algorithms J Dairy Sci 2010 93 2229 2238 10.3168/jds.2009-2849 20412938

[B6] Zhang Z Druet T Marker imputation with low-density marker panels in Dutch Holstein cattle J Dairy Sci 2010 93 5487 5494 10.3168/jds.2010-3501 20965364

[B7] Scheet P Stephens M A fast and flexible statistical model for large-scale population genotype data: Applications to inferring missing genotypes and haplotypic phase Am J Hum Genet 2006 78 629 644 10.1086/502802 16532393 PMC1424677

[B8] Browning BL Browning SR A unified approach to genotype imputation and haplotype phase inference for large data sets of trios and unrelated individuals Am J Hum Genet 2009 84 210 223 10.1016/j.ajhg.2009.01.005 19200528 PMC2668004

[B9] VanRaden PM O'Connell JR Wiggans GR Weigel KA Combing different marker densities in genomic evaluation Interbull Bull 2010

[B10] McCarthy MI Abecasis GR Cardon LR Goldstein DB Little J Ioannidis JP Hirschhorn JN Genome-wide association studies for complex traits: consensus, uncertainty and challenges Nature Reviews: Genetics 2008 9 356 369 10.1038/nrg2344 18398418

[B11] Goddard ME Hayes BJ Mapping genes for complex traits in domestic animals and their use in breeding programmes Nature Reviews: Genetics 2009 10 381 91 10.1038/nrg2575 19448663

[B12] Jiang L Liu J Sun D Ma P Ding X Ying Y Zhang Q Genome wide association studies for milk production traits in Chinese Holstein population PloS One 2010 5 10 907 920 10.1371/journal.pone.0013661 PMC2965099 21048968

[B13] Schaeffer LR Jamrozik J Kistemaker GJ Van Doormaal BJ Experience with a test-day model J Dairy Sci 2000 83 1135 1144 10.3168/jds.S0022-0302(00)74979-4 10821590

[B14] Madsen P Srensen P Su G Damgaard LH Thomsen H Labouriau R DMU-A package for analyzing multivariate mixed models. Proceedings of the 8th World Congress on Genetics Applied to Livestock Production Belo Horizonte, Minas Gerais, Brazil 2006 13 18

[B15] Zhang Z Liu J Ding X Bijma P de Koning DJ Zhang Q Best linear unbiased prediction of genomic breeding values using a trait-specific marker-derived relationship matrix PloS One 2010 5 e12648 10.1371/journal.pone.0012648 20844593 PMC2936569

[B16] Hayes BJ Bowman PJ Chamberlain AJ Goddard ME Invited review: genomic selection in dairy cattle: progress and challenges J Dairy Sci 2009 92 433 443 10.3168/jds.2008-1646 19164653

